# Evaluating the Potential of *Boswellia rivae* to Provide Sustainable Livelihood Benefits in Eastern Ethiopia

**DOI:** 10.3390/plants12102024

**Published:** 2023-05-18

**Authors:** Anjanette DeCarlo, Stephen Johnson, Abdinasir Abdikadir, Prabodh Satyal, Ambika Poudel, William N. Setzer

**Affiliations:** 1 The Aromatic Plant Research Center, 230 N 1200 E, Suite 100, Lehi, UT 84043, USA; anjanettedecarlo@gmail.com (A.D.); psatyal@aromaticplant.org (P.S.);; 2 Grossman School of Business, University of Vermont, 55 Colchester Ave, 100 Kalkin Hall, Burlington, VT 05405, USA; 3 FairSource Botanicals, LLC, 560 Fox Drive #643, Fox Island, WA 98333, USA; 4 Somali Region Pastoral and Agro-Pastoral Research Institute, Jigjiga P.O. Box 1020, Ethiopia; 5 Department of Chemistry, University of Alabama in Huntsville, 301 Sparkman Drive, Huntsville, AL 35899, USA

**Keywords:** *Boswellia rivae*, chemical composition, rural livelihoods, frankincense, *Commiphora africana*, Ogaden

## Abstract

Frankincense is an oleo-gum-resin collected from wild *Boswellia* spp. trees, and widely used in perfumery, cosmetics, aromatherapy, incense, and other industries. *Boswellia rivae*, growing in Ethiopia, Somalia, and Kenya, is one source of frankincense, but is little-commercialized compared to species such as *B. sacra*, *B. frereana*, and *B. papyrifera*. In this study, we examine the resin essential oil chemistry and harvesting systems of *B. rivae* in order to evaluate its potential for increased trade and potential positive livelihood benefits. *Boswellia rivae* produces an essential oil rich in α-thujene (0.1–12.4%), α-pinene (5.5–56.4%), β-pinene (0.3–13.0%), δ-3-carene (0.1–31.5%), *p*-cymene (1.4–31.2%), limonene (1.8–37.3%), β-phellandrene (tr-5.6%), *trans*-pinocarveol (0.1–5.0%), *trans*-verbenol (0.1–11.2%), and *trans*-β-elemene (0–5.7%), similar to major commercial species, although it is difficult to detect mixing of *B. rivae* and *Commiphora africana* resins from chemistry alone. The *B. rivae* trees are not actively tapped, so resin collection has a neutral impact on the health of the trees, and resin production is unaffected by drought. Consequently, collecting resins acts as a key income supplementing livestock herding, as well as a safety net protecting pastoral communities from the severe negative effects of climate change-exacerbated drought on livestock. Therefore, *Boswellia rivae* is well positioned chemically, ecologically, and socially to support expanded trade.

## 1. Introduction

The collection of wild plants provides numerous benefits, including food, medicine, cordage, construction materials, and income from onwards sale of collected materials. Most of these wild plants count as nontimber forest products (NTFPs), a broad term that is generally understood to mean biological materials other than timber collected from ecosystems and that provide local benefits [1,2]. The global income from NTFPs was estimated at USD 88 billion in 2011 [3]. NTFPs were initially heralded as a way to both produce significant income to support local community development and to incentivize the conservation of ecosystems through these benefits [4,5,6]. However, the growing body of NTFP research over the past 30 years has shown that the socioecological systems in which NTFPs are produced, and the relationships between NTFPs, their ecosystems, the collectors/harvesters, and the markets that purchase these NTFPs, have more complex and subtle dynamics than previously appreciated [2]. As a result, the link between commercialization and positive socioecological benefits is not always straightforward [7]. Still, NTFPs play a critical role in the livelihoods of communities (particularly those of the poorest members of those communities) around the world, often representing between 10–50% of total household income; in some instances, that share may be even higher [8,9,10]. This income serves to support daily provisioning and household expenses, allow for greater savings of cash for other uses, and, critically, can provide a safety net of income should other livelihood activities be affected [11,12]. NTFPs thus often play a critical role in poverty prevention, mitigation, and/or alleviation, and can be important vehicles to support development and community stability, especially if they are already successfully commercialized.

Frankincense is one such already commercialized NTFP. It is an aromatic terpenoid oleo-gum-resin, produced by trees in the genus *Boswellia* Roxb. ex Colebr. (Burseraceae: Sapindales). The genus includes approximately 24 species widely distributed across Sahelian west Africa to the Horn of Africa, the southern Arabian Peninsula, and through much of the Indian subcontinent [13]. Perhaps 8–9 species are harvested in significant quantities for international trade [14]. *Boswellia* species are small to medium size trees, typically characterized by monoecious flowers, imparipinnate compound leaves, papery, exfoliating bark, and a dark red resiniferous layer of inner bark containing resin canals, in which the frankincense resin is produced and stored. The resin serves to protect the trees from minor insults, insect attacks, and pathogens, and exudes when the bark is broken and the resin canals are breached [13]. Many *Boswellia* species are experiencing documented or suspected population declines, due to a variety of factors such as ungulate (primarily camels, goats, and cattle) grazing, overharvesting of resin, fire, insect attacks, and land conversion for agriculture (in suitable areas) [15,16,17,18]. These threats and the consequent status of *Boswellia* populations is, for the major commercial species, tied to the commercialized value of the frankincense resin, both in positive and negative ways: excessive/inappropriate resin tapping causes damage to the trees, which in turn increases their susceptibility to insect attacks, but the value of the resin can in some cases also protect trees from land clearance for agriculture or from excessive ungulate grazing [14,19]. The relationship between commercialization and sustainability in *Boswellia* species is complex, depending on both the local circumstances of collection, and the structure of incentives within the commercial value chain [14,19]. 

Frankincense is one of the oldest internationally traded commodities, with sophisticated supply chains established more than 2000 years ago [20]. The resins are primarily used in perfumery, cosmetics, aromatherapy, naturopathic supplements, traditional medicinal markets (Chinese traditional medicine, Ayurveda, etc.), incense production, and as chewing gum [14]. While there is some crossover, these different markets prefer certain *Boswellia* species over others, following chemical differences in the resin. *Boswellia papyrifera* is primarily used for incense and boswellic acid extraction for supplements due to its low yield of octyl acetate and octanol-dominated essential oil; by contrast, *Boswellia sacra* yields higher levels of essential oil composed of monoterpenes, such as α-pinene, limonene, and myrcene, and is preferred by the perfumery, cosmetics, and aromatherapy industries [14,21,22].

Frankincense supply chains are generally nontransparent, with a series of brokers or middle traders and relatively low prices paid directly to harvesters [18]. Collection is seasonal, typically taking place during the dry season, and many harvesters follow a mixed livelihood strategy, combining frankincense and other NTFP harvesting with livestock herding or other activities [23,24]. The frankincense trade is typically highly segmented by gender, with men harvesting the resin and women sorting it to remove bark and other impurities. While frankincense from many species has been internationally traded for millennia, two recent developments have prompted increased interest in the sustainability of sourcing frankincense: first, a significant increase in the demand for frankincense essential oil, spurred by the aromatherapy industry, and second, a series of studies indicating social concerns, unsustainable practices, and actual or potential population declines in some of the major commercial species [15,16,17,18,25]. Concerns over sustainability have also led some companies to use blends of multiple species, or to investigate the use of alternative species [14]. 

*Boswellia rivae* is one species that holds promise as an ‘alternative frankincense’. Growing abundantly in the Somali Region of eastern Ethiopia as well as Somalia and northeastern Kenya, *B. rivae* is already traded in modest amounts for incense, perfume, and aromatherapy, with established commercial supply chains and collection practices [13,14]. Although the composition of commercial samples of *B. rivae* has previously been reported, there is confusion as to whether these samples are pure or represent a mixture of species indiscriminately collected alongside *B. rivae* [14]. In addition to being listed as least concern on the 2018 IUCN Red List of Threatened Species, it is one of two (existing) commercial species that is not actively tapped, but instead is reported to have its resin collected exclusively from natural self-exudations [26]. As a result, collection has far less impact on the trees than in other species of actively tapped frankincense such as *B. papyrifera* and *B. sacra*. Planning and preparing a sustainable collection system is an involved process beyond the scope of a single paper, including resource inventories, yield and regeneration studies, etc. However, before investing the resources to carry out these studies, it is important to know there is market potential. In this study, we aim to provide first reporting of confirmed pure samples of *B. rivae* and the potential adulterant species *Commiphora africana*, compare the essential oil of *B. rivae* to other commercial species, trace the supply chain, understand how it currently contributes to local livelihoods, and evaluate the potential for expanded production to contribute to rural income in southeastern Ethiopia. 

## 2. Results

### 2.1. Chemical Composition of Resin Samples

The essential oils from the three *B. rivae* direct tree samples, two *C. africana* direct tree samples, and five commercial samples sold as *B. rivae* were obtained using hydrodistillation with yields of 8.74–14.53% (*B. rivae; w*/*w*), 10.76–23.55% (*C. africana; w*/*w*), and 3.57–13.96% (commercial; *w/w*), respectively, as yellow oils (Table 1). The essential oil compositions are compiled in Table 2. The compositions were all fairly similar, dominated by monoterpenes (51.5–82.8%) and oxygenated monoterpenoids (14.9–37.1%), with low (typically <5%) concentrations of sesquiterpenes, oxygenated sesquiterpenes, diterpenoids, benzenoid aromatics, and other components. 

The major components of the essential oils were similar across all samples, including between *B. rivae* and *C. africana*. Major components included α-thujene (0.1–12.4%), α-pinene (5.5–56.4%), β-pinene (0.3–13.0%), δ-3-carene (0.1–31.5%), *p*-cymene (1.4–31.2%), limonene (1.8–37.3%), β-phellandrene (tr-5.6%), *trans*-pinocarveol (0.1–5.0%), *trans*-verbenol (0.1–11.2%), and *trans*-β-elemene (0–5.7%). A small number of components were present in both of the two pure *C. africana* samples but not any of the three pure *B. rivae* samples (Table 1). These include toluene, 6,6-dimethylhepta-2,4-diene, tricyclene, 4-methylpent-2-enolide, 1,3,5-trimethylcycloheptane, *p*-menth-1-ene, trimethylbicyclo[2.2.1]hept-5-en-2-one, chrysanthenone, *cis*-p-menth-2-en-1-ol, nopinone, *cis*-pinocamphone, filifolide A, (*Z*)-β-ocimene, and caryophyllene oxide. The abundance of these components is low in all cases (tr—0.3%).

Chiral GC–MS analysis indicated that α-thujene, α-copaene, and *trans*-β-elemene were exclusively levorotary, while δ-3-carene, β-thujone, and verbenone were exclusively dextrorotary across all samples. Sabinene and camphene were largely levorotary, while α-phellandrene was almost exclusively dextrorotary except for a single sample. α-Pinene, β-pinene, and limonene showed highly variable enantiomeric distributions between samples. No clear patterns were apparent between *B. rivae* and *C. africana* samples (Table 3).

**Figure 1:**
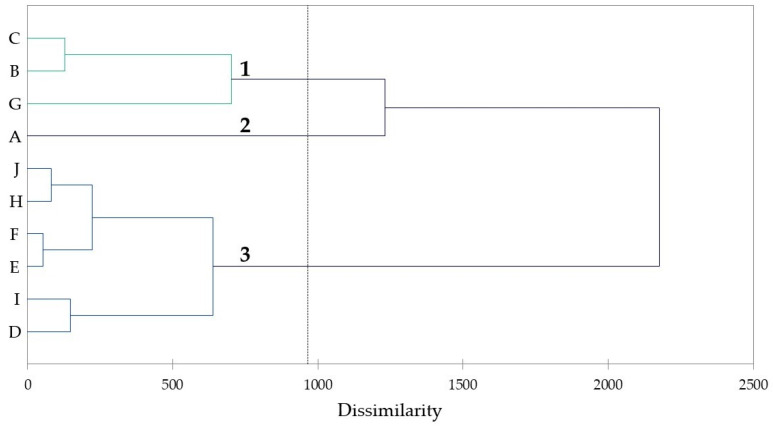
Agglomerative hierarchical cluster (AHC) analysis based on the concentrations of chemical constituents.

A hierarchical cluster analysis indicated that the samples fell into three distinct compositional clusters: a limonene/α-pinene group (cluster #1), a single sample dominated by δ-3-carene (cluster #2), and a group dominated by α-pinene, occasionally with significant spikes of *trans*-verbenol, *p*-cymene, or β-pinene (cluster #3) (Figure 1).

### 2.2. Harvester Perceptions and Collection System

The harvesters interviewed unanimously stated that the resins are collected only when they naturally exude from the tree, with no active tapping taking place. This was confirmed using field observations, where no active tapping was apparent even near harvesting villages. Interestingly, the harvesters reported that collection of resin resources is often segmented by gender, with women collecting *B. rivae* resins and men collecting resins of *Commiphora myrrha* and *C. guidottii*, reportedly as the prominent spines of the latter species make collection difficult for women wearing the standard long, flowing dresses. However, during periods of higher demand, these gender divides do not apply as strictly and both genders collect all types of resin. Additionally, children may accompany their adult family members on collecting trips, although this was not said to be common. In addition to being collected for trade, resins were reportedly burned locally to purify spaces and heal sick children, chewed, and applied topically to heal wounds.

All harvesters interviewed agreed that only a small amount of the resin produced is currently collected and traded, with the opportunity for significantly expanded collection if there is a market. No harvesters perceived issues with sustainability or declines in tree health or abundance. Although drought has been impacting the region for several years, informants did not perceive a negative impact on the health of the *B. rivae* trees; to the contrary, they perceived the drought to increase resin production. Resin collection was also reported to be one of the only good sources of income during the drought, due to the negative effects of drought on livestock, and collection seems to be expanded during periods of drought. However, the response to drought was not uniform, as harvesters from one village reported a decrease in resin collection during drought as a result of most people spending increased time trying to save their livestock.

**Figure 2:**
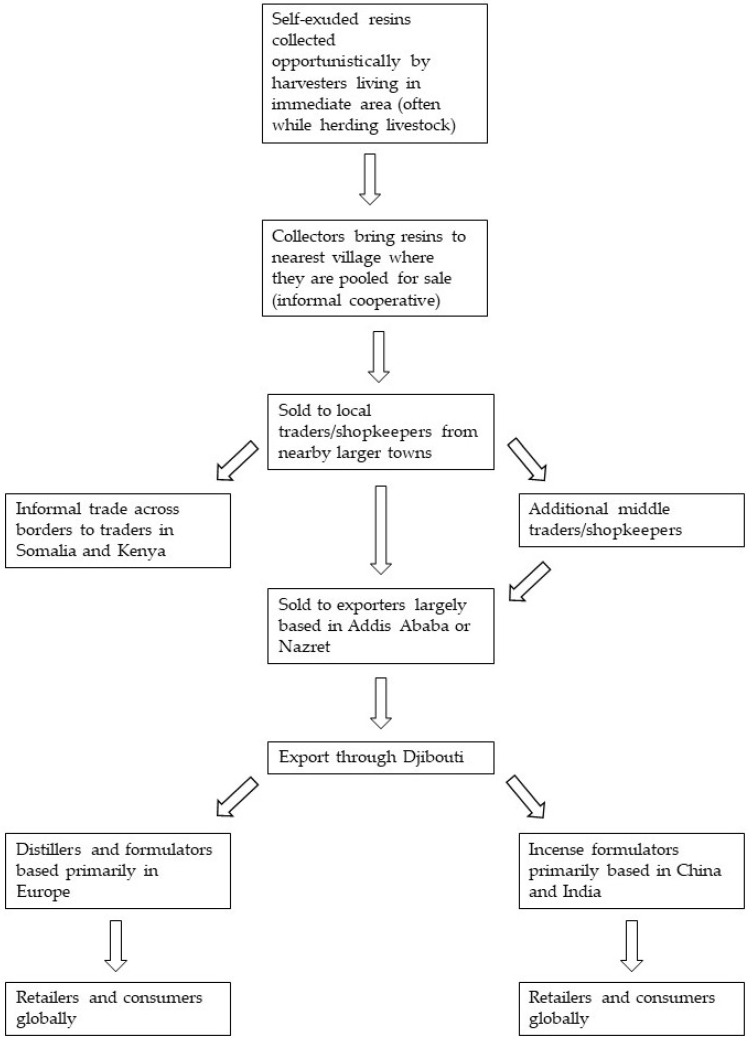
Diagram of *B. rivae* resin supply chain in southern Ogaden region, Ethiopia.

Resins are collected opportunistically, predominantly by women, with harvesters sometimes making dedicated collecting trips and sometimes collecting resins as they herd livestock, and then brought back to the harvesters’ village for storage. Harvesters from a given village reported pooling their resins for sale to local traders, in practice forming an informal sales cooperative. These traders in turn sell the resins to national exporters who export the resins to processors, largely based in Europe (Figure 2). Harvesters reported that the sales price of the resin fluctuates seasonally and annually, due to market changes, exchange rate changes, and the quality (size, color, percentage foreign bodies in the resin) of the resin but is normally USD 1–1.25 per kg of resin. As a focused harvester can collect several hundred kilograms of resin per year, this represents a significant source of income for harvesting communities.

## 3. Discussion

### 3.1. Essential Oil Composition and Market Potential

Both the direct tree and commercial samples of *B. rivae* show a similar composition to major commercial species of frankincense. *Boswellia sacra* (syn. *B. carteri*), *B. frereana*, and *B. serrata* represent the primary source of frankincense essential oil on the commercial market. *Boswellia sacra* is known primarily for its α-pinene-rich essential oil, although an α-thujene-dominant chemotype has also been reported [27,28,29,30]. It is also one of the most variable species, with multiple subgroups within the overall α-pinene chemotype: (1) an α-pinene/limonene group, (2) a limonene/α-pinene group, and (3) a group variably dominated by myrcene, sabinene, limonene, α-pinene, viridiflorol, β-caryophyllene, and/or *p*-cymene [27]. *Boswellia frereana* essential oil contains varying levels of α-thujene and α-pinene, with moderate amounts of sabinene, *p*-cymene, and α-phellandrene dimers [31,32]. *Boswellia serrata* essential oil, by contrast, is dominated by α-thujene, with minor components including myrcene, methyl eugenol, methyl chavicol, sabinene, kessane, and α-pinene [33].

The *B. rivae* samples collected in this study have a similar composition to *B. sacra*, the most popular commercial essential oil. There are some exceptions, with one commercial sample showing an unusually high level of *p*-cymene and one tree sample showing a high level of δ-3-carene with very little α-pinene; the *B. rivae* samples are also typically higher in *trans*-verbenol than most *B. sacra* [27,28,29,30]. However, this is not necessarily a barrier to further commercialization, as individual deviant samples can be blended into batches of resin with the more common and preferred α-pinene/limonene-dominant profiles. Additionally, *B. rivae* can be distinguished from *B. sacra*, as it lacks incensole or incensyl acetate; it can also be distinguished from the much rarer conspecific *B. ogadensis* by the absence of 3,5-dimethoxytoluene and (*Z*)- and/or (*E*)-salvene [34]. These chemical markers would allow the authentication of *B. rivae* as a non-tapped, sustainably collected source of frankincense.

Mixing of resins from multiple species was acknowledged as common practice by many of the harvesters interviewed. Most commonly, this involved collection of both *Boswellia rivae* and *Commiphora africana*, as the two resins are highly similar in both appearance (see Figure 1) and scent. Indeed, the essential oil profiles of the *C. africana* samples were essentially indistinguishable from those of *B. rivae*. A number of minor components that may act as markers of *C. africana* were identified, although they would likely only be apparent if the percentage of *C. africana* in the resin were relatively high; this will need to be confirmed with further study. This difficulty in establishing clear species identification may be a barrier to expanded commercialization, as industries typically prefer clear, single-species product identification as a means of enhancing quality control and meeting regulatory obligations. However, from a local perspective, the logic of mixed species collection makes sense as long as traders do not raise an issue with it, as this allows expanded product collection with less effort. The chemical similarity between multiple species makes imposing postcollection, species-based quality checks difficult, and without this kind of control, there is little incentive for harvesters to adhere to single species collection.

### 3.2. Collection System and Role in Local Livelihoods

*Boswellia rivae* is unusual amongst frankincense species, and resin-bearing species in general, for being passively harvested. Most species are actively tapped to obtain the resin, necessarily causing damage to the harvested tree and presenting opportunities for pests and pathogens to attack the tree [13,35]. Active tapping also affects the carbon resources of the tree, reducing reproduction and growth [36,37]. Harvesting pressure driven by the expanding market for frankincense has affected other species of *Boswellia*, particularly *B. sacra* in Somaliland, where higher demand for resin combined with limited market incentives for sustainability have resulted in a wave of unsustainable harvesting practices [18]. *Boswellia rivae*, on the other hand, is relatively insulated from the effects of a potential market wave, as resin collection has a neutral impact on the trees. This positions the species well to support expanded market demand without negative ecological impacts. *Boswellia neglecta* is the only other non-tapped frankincense species, but its essential oil contains high levels of undesirable components such as terpinen-4-ol, limiting its market potential [38]. By contrast, *B. rivae* has relatively low levels of terpinen-4-ol, but often significant quantities of commonly desirable components such as α-pinene and limonene.

Although some harvesters reported collecting less resin during times of drought as a result of spending more time attempting to keep livestock alive, the trees’ production of the resins was reportedly unaffected by drought conditions, representing a potential safety net of income even during livestock die-offs [24,39,40]. Few other alternative sources of income exist in these areas, so the collection of resins is a critical source both of supplementary income and stability, especially as climate change is likely to drive increasing frequency and severity of drought [41,42]. The collection of resins is generally considered culturally inferior to income from herding livestock, but it is a key source of cash resilient to the impacts of drought [18]. The involvement of women as direct collectors of the resins is also unusual in frankincense, where most species’ collection systems follow a rigid traditional system of men harvesting and women sorting/cleaning the resins. This is particularly important given Ethiopia’s low human development classification and pervasive gender income inequality [43,44]. The collection and sale of NTFPs by women has been effective at reducing gender-based income inequality elsewhere in Ethiopia, highlighting the opportunity for expanded commercialization in *B. rivae* to follow a similar pattern [45].

## 4. Materials and Methods

### 4.1. Study Species

*Boswellia rivae* Engl. is one of 24 species of *Boswellia*, and one of six known from Ethiopia. It is distributed widely in eastern Ethiopia, Somalia, and northeastern Kenya, where it prefers *Acacia*-*Commiphora* woodland at elevations of 150–915 m above sea level. It was most recently assessed as least concern under the IUCN Red List Categories and Criteria in 2018 and has not been reported to be under significant threat. The species grows both on flat areas and on rocky slopes, in a variety of substrates including limestone, gypsum, and sandy soil. It grows as a small tree or spreading shrub, with dark yellow to pale grey exfoliating bark, imparipinnate leaves 4–18 cm long, with slightly serrated leaflets. Flowers are pink, occurring in pubescent racemes or panicles up to 6 cm long. The fruits are angular, pyriform, and pubescent, 3(-4)-locular, with pyrenes up to 10 × 7 mm [13].

The species is well known locally where it occurs, with both the tree and resin called Mirafur; sometimes other names are applied as well, such as beeyo or jawder, but these more properly refer to *B. sacra* and *B. neglecta*, respectively, and are rarely used to describe *B. rivae*. The resin is collected and used locally for incense, chewing, and as insect repellent. The wood is also reportedly used in construction and fencing, although the generally twisted branching architecture and softness of the wood do not make it ideal for this purpose [13].

### 4.2. Study System

The Somali Region of Ethiopia, also known as the Ogaden, is a large (~327,000 km^2^) regional state consisting of an uplifted plateau sloping from about 1500 m in the northwest to 300 m in the south. The soils consist primarily of sedimentary limestone formed during the cretaceous and lower cretaceous periods. They are typically rich in potassium, phosphorus, and carbonates, but are low in nitrogen, and are fertile only with irrigation [46]. Annual precipitation varies from 200–400 mm, with a dual rainy season system that produces most of the precipitation during April–May and September–October [47]. *Acacia*-*Commiphora* woodland is dominant through most of the Ogaden region, grading into desert and semi-desert scrubland at lower elevations in the south of the region [48]. Species of *Vachellia* spp., *Senegalia* spp., *Commiphora* spp., *Boswellia* spp., *Balanites aegyptiaca*, and *Maytenus senegalensis* are common [49].

### 4.3. Interviews with Harvesting Communities

Field surveys of *B. rivae* production sites in southeastern Ethiopia took place in June 2022, focusing on known harvesting villages. A total of 28 participants (8 women, 20 men) were interviewed using participatory semi-structured and narrative interviews, focusing on local peoples’ perceptions of resource abundance, livelihood benefits, trade structure, local uses, and impacts of disturbances like drought. A semi-structured and narrative format was used to allow participants to raise key issues themselves and indicate the issues they found most important and impactful. Key pieces of information were authenticated using triangulation with at least three independent informants.

### 4.4. Collection of Resins

**Figure 3:**
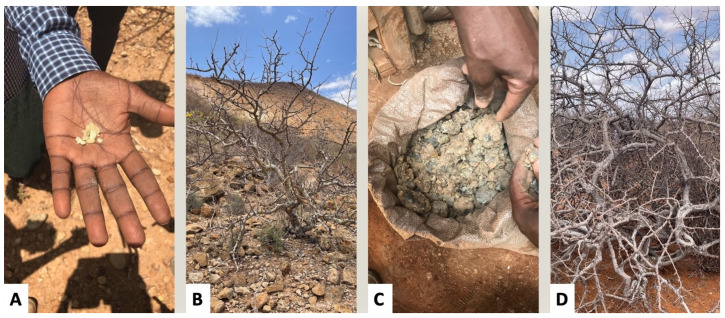
Fresh *B. rivae* resin collected directly from the tree (**A**); *B. rivae* tree in situ (**B**); *B. rivae* resin is often mixed with soil due to being collected from the ground under the trees (**C**); *Commiphora africana* tree in situ (**D**). As can be seen, it can be difficult to distinguish *B. rivae* from *C. africana* during the dry seasons when the leaves have dropped.

Five samples of resins said to be *B. rivae* were collected from harvesting villages and commercial stores, which served as examples of *B. rivae* resin currently in trade. As a control, three samples were also collected directly from *B. rivae* trees and two samples directly from *Commiphora africana* trees (locally called Geed Harag, and sometimes collected along with the *B. rivae*); these control samples allowed us to confirm the botanical identity of the trees, examine pure samples of these species’ essential oils, and compare these pure samples to the village/commercial samples (Figure 3). This allowed us to examine if components occurring in pure *C. africana,* but not pure *B. rivae,* also appeared in the commercial samples and thus indicated mixing of *B. rivae* and *C. africana* in the commercially-traded samples. Given the small amount of resin produced per tree (often only a few grams), we pooled resins from individual trees in a given area, such that each sample represents resins taken from multiple trees in a given sample location. The resins collected were naturally exuded, and thus of varying ages, but we focused on newly exuded resins and did not collect clearly old and excessively dry resins. Resins were sealed in plastic bags and shipped to the Aromatic Plant Research Center for analysis. Voucher specimens of both *B. rivae* and *C. africana* were deposited at the Jigjiga Herbarium at the Somali Region Pastoral and Agro-Pastoral Research Institute (*B. rivae*, specimen no. 7205; *C. africana*, specimen no. 7206) and identified by A.A.

### 4.5. Hydrodistillation of Resins

Hydrodistillation of the resin samples was carried out using a Likens–Nickerson apparatus [50] with continuous extraction with dichloromethane for 6 h each to provide yellow to pale yellow essential oils (Table 3). For each hydrodistillation, the resin was placed in a 500 mL flask with 200 mL of distilled water, the Likens–Nickerson apparatus suitable for solvents heavier than water was used [51], 25 mL of dichloromethane was placed in a 50 mL round-bottom flask for the continuous extraction of the hydrodistillate. The condenser was maintained at 10–15 °C with a recirculating refrigerated water bath. The hydrodistillation was carried out under normal atmosphere.

### 4.6. Gas Chromatography-Mass Spectrometry

The *B. rivae* and *C. africana* resins were analyzed using GC–MS with a Shimadzu GCMS-QP2010 Ultra (Shimadzu Scientific Instruments, Columbia, MD, USA) with ZB-5ms capillary column (Phenomenex, Torrance, CA, USA), as previously described [27]. Identification of the chemical components was carried out by comparison of the retention indices determined with respect to a homologous series of normal alkanes and our comparison of their mass spectra with those reported in the literature [52] and the Aromatic Plant Research Center’s inhouse library [53].

### 4.7. Gas Chromatography-Flame Ionization Detection

The *B. rivae* and *C. africana* oleogum resin essential oils were analyzed using GC–FID using a Shimadzu GC 2010 (Shimadzu Scientific Instruments, Columbia, MD, USA) equipped with flame ionization detector, a split/splitless injector, and Shimadzu autosampler AOC-20i (Shimadzu Scientific Instruments, Columbia, MD, USA), with a ZB-5 capillary column (Phenomenex, Torrance, CA, USA), as previously described [27]. Percent compositions were determined using peak integration without standardization.

### 4.8. Chiral Gas Chromatography-Mass Spectrometry

The *B. rivae* and *C. africana* essential oils were analyzed using chiral GC–MS as previously reported [54]: Shimadzu GCMS-QP2010S instrument (Shimadzu Scientific Instruments, Columbia, MD, USA), Restek B-Dex 325 capillary column (30 m × 0.25 mm × 0.25 μm film) (Restek Corporation, Bellefonte, PA, USA). Enantiomers of monoterpenoids identified by comparison of retention times with standards (Sigma-Aldrich, St. Louis, MO, USA) and percentages determined based on peak areas.

### 4.9. Hierarchical Cluster Analysis

The essential oil compositions for each sample were treated as operational taxonomic units (OTUs), and the percentages of the most abundant essential oil components (α-pinene, limonene, *p*-cymene, β-pinene, *trans*-verbenol, α-thujene, δ-3-carene, *trans*-pinocarveol, verbenone, *trans*-β-elemene, sabinene, *trans*-carveol, α-terpineol, terpinen-4-ol, *p*-cymen-8-ol, myrtenol, β-phellandrene, α-copaene, myrtenal, linalyl acetate, α-phellandrene, and α-phellandrene dimers) were used to establish chemical associations between the essential oil samples using agglomerative hierarchical cluster (AHC) analysis using XLSTAT Premium, version 2018.1.1.62926 (Addinsoft, Paris, France). Dissimilarity was determined using Euclidean distance, and clustering was defined using Ward’s method with automatic entropy truncation.

## 5. Conclusions

*Boswellia rivae* produces an essential oil chemical composition comparable to that of other major commercial species, although it has not yet been commercialized to the same degree. The mixing of non-frankincense resins into the *B. rivae* collections is a concern, but the essential oil profiles of *B. rivae* and *C. africana* are similar, and commercial samples, regardless of mixing, are comparable to major traded frankincense species such as *B. sacra*. Given the neutral impact of resin collection on the species due to the passive collection system, and the least concern status of the species, trade in *B. rivae* could likely be expanded significantly without negative ecological consequences for the species. This would also have strong positive social benefits, as resin collection is a critical supplementary livelihood and bulwark against the increasing effects of climate change-driven drought. Therefore, expanding resin collection and trade would likely function as a critical support to pastoral communities under environmental pressure.

## Figures and Tables

**Table 1:** Resin collection and hydrodistillation details.

Sample	Collection Site	Mass Resin	Essential Oil Yield (*w*/*w*)
A	*B. rivae* tree sample A: 5°16′35.41″ N, 43°30′34.00″ E, 518 m asl	37.59 g	14.53%
B	*B. rivae* tree sample B: 5°51′5.99″ N, 43°50′1.77″ E, 363 m asl	24.64 g	12.49%
C	*B. rivae* tree sample C: 6°0′18.08″ N, 44°47′10.18″ E, 391 m asl	30.48 g	8.74%
D	Commercial sample D: Qarsodi, 6°24′30″ N, 44°42′32.4″ E	31.36 g	10.16%
E	Commercial sample E: Higloley, 5°45′58.8″ N, 44°33′49.2″ E	37.81 g	3.57%
F	Commercial sample F: Kebri Dehar, 6°44′27.348″ N, 44°16′17.569″ E	32.20 g	13.96%
G	Commercial sample G: Jigjiga, 9°21′18.677″ N, 42°48′7.981″ E	30.88 g	11.43%
H	Commercial sample H: Shilabo, 6°5′18.582″ N, 44°45′52.362″ E	31.31 g	9.07%
I	*C. africana* tree sample I: 5°16′35.47″ N, 43°30′34.13″ E, 519 m asl	25.93 g	10.76%
J	*C. africana* tree sample J: 5°45′58.8″ N, 44°33′49.2″ E, 475 m asl	26.62 g	23.55%

**Table 2:** Chemical composition (%) of *B. rivae* and *C. africana* resin essential oils.

RI_calc_	RI_db_	Compound	A	B	C	D	E	F	G	H	I	J
782	782	Toluene	---	---	---	---	---	---	---	---	0.1	0.1
834	847	6,6-Dimethylhepta-2,4-diene	---	---	---	---	---	---	---	---	0.2	0.1
847	846	(*Z*)-Salvene	---	---	---	---	---	---	---	---	---	0.1
919	919	Hashishene	0.1	tr	0.1	tr	tr	tr	0.1	tr	0.1	tr
923	923	Tricyclene	---	---	---	---	---	---	---	---	tr	0.1
925	927	α-Thujene	12.4	1.6	4.5	0.3	2.1	3.1	2.3	2.3	0.1	8.6
933	933	α-Pinene	5.5	30.4	31.9	45.1	24.2	25.7	16.6	39.4	56.4	35.0
942	942	4-Methylpent-2-enolide	---	---	---	---	0.1	0.1	tr	0.1	0.2	0.1
948	948	α-Fenchene	0.1	---	tr	tr	tr	0.1	tr	tr	0.1	tr
949	950	Camphene	0.1	0.4	0.4	0.3	0.6	0.8	0.3	0.6	0.6	0.7
953	953	Thuja-2,4(10)-diene	---	0.1	0.1	0.1	0.2	0.2	0.1	0.3	0.6	0.2
954	955	3-Methylapopinene	---	---	tr	---	tr	tr	tr	---	---	---
965	964	3,7-Dimethyl-2-octene	---	---	---	---	---	0.1	---	---	---	---
970	970	3,7,7-Trimethyl-1,3,5-cycloheptatriene	1.7	0.1	---	---	---	---	---	0.3	---	---
972	972	Sabinene	1.3	0.8	1.1	2.6	1.2	1.6	0.4	1.1	0.2	3.7
978	978	β-Pinene	0.3	1.5	3.1	10.9	6.2	9.5	1.5	8.5	5.2	13.0
984	982	6-Methylhept-5-en-2-one	---	---	---	---	---	---	tr	tr	---	---
987	987	*p*-Menth-3-ene	---	---	---	---	---	---	0.1	---	---	---
989	989	Myrcene	---	0.1	0.2	0.9	tr	tr	0.1	---	tr	---
990	990	Dehydro-1,8-cineole	---	---	0.1	tr	0.1	tr	---	tr	---	---
990	989	1,3,5-Trimethylcycloheptane	---	---	---	---	---	---	---	---	0.1	0.1
993	995	6-Methylhept-5-en-2-ol (= Sulcatol)	---	---	tr	tr	---	---	0.1	---	---	---
999	1000	δ-2-Carene	0.1	---	---	---	---	---	---	---	---	---
1001	1000	*p*-Menth-2-ene	---	---	---	---	---	0.2	---	---	---	---
1004	1004	*p*-Mentha-1(7),8-diene	0.2	0.2	0.2	0.1	0.1	tr	0.2	---	0.1	---
1006	1006	3-Ethenyl-1,2-dimethyl-1,4-cyclohexadiene	---	---	---	---	---	---	---	---	0.1	---
1007	1007	α-Phellandrene	---	---	---	3.4	0.3	0.3	1.3	---	---	---
1009	1009	2-Methylanisole	---	0.1	0.2	---	0.1	0.1	0.2	0.1	0.4	---
1010	1009	δ-3-Carene	31.5	0.6	0.6	0.1	0.6	0.3	0.6	0.8	0.2	0.2
1018	1018	α-Terpinene	0.1	tr	tr	0.2	tr	tr	0.1	tr	0.1	0.1
1020	1022	*m*-Cymene	0.7	0.1	0.1	tr	0.2	0.2	0.1	0.2	tr	0.3
1023	1023	*p*-Menth-1-ene	---	---	---	---	---	---	---	---	0.1	0.3
1025	1025	*p*-Cymene	9.8	1.8	13.1	1.7	8.4	6.2	31.2	3.7	1.4	2.7
1026	1026	2-Acetyl-3-methylfuran	0.3	---	0.1	tr	0.4	0.5	---	0.3	---	0.8
1030	1030	Limonene	16.5	37.3	27.1	9.3	11.2	3.0	16.9	1.9	5.4	1.8
1032	1031	β-Phellandrene	0.2	tr	0.3	5.6	0.2	0.1	1.3	tr	0.1	tr
1032	1032	1,8-Cineole	0.1	tr	0.1	0.1	0.1	0.1	0.2	tr	tr	tr
1035	1034	(*Z*)-β-Ocimene	---	---	---	0.1	---	---	0.1	---	tr	tr
1036	1039	*o*-Cymene	0.2	0.2	0.1	---	0.2	0.2	---	0.1	0.3	0.2
1046	1045	(*E*)-β-Ocimene	---	---	---	0.1	---	---	---	---	---	---
1058	1058	γ-Terpinene	0.1	tr	tr	0.3	0.1	0.1	0.1	0.1	0.1	0.1
1070	1069	*cis*-Sabinene hydrate	0.1	0.1	0.1	0.1	0.3	0.5	0.1	0.4	0.1	0.2
1071	1069	*cis*-Linalool oxide (furanoid)	---	---	---	tr	---	---	---	---	---	---
1072	1072	*p*-Cresol	0.1	---	0.1	---	0.1	0.1	0.1	tr	---	---
1081	1080	*m*-Cymenene	0.1	---	---	---	---	---	---	---	---	---
1086	1086	Neral	---	---	---	---	0.6	0.6	---	---	---	---
1087	1086	*trans*-Linalool oxide (furanoid)	---	---	---	0.2	---	---	---	---	---	---
1087	1087	Terpinolene	0.1	---	---	0.2	---	---	---	---	---	---
1090	1091	*p*-Cymenene	0.1	---	0.1	tr	0.2	---	0.3	0.1	---	---
1096	1099	6-Camphenone	0.1	---	---	---	---	---	---	---	---	---
1098	1097	α-Pinene oxide	---	0.2	---	---	---	0.1	---	---	tr	0.1
1100	1101	Linalool	0.1	---	0.1	0.9	0.1	---	0.1	---	---	---
1101	1101	*trans*-Sabinene hydrate	0.1	0.1	0.1	tr	0.4	0.5	0.1	0.3	0.1	0.2
1102	1103	1,7,7-Trimethylbicyclo[2.2.1]hept-5-en-2-one	---	---	---	---	---	---	---	---	0.1	0.1
1104	1104	Hotrienol	---	---	---	tr	---	---	---	---	---	---
1107	1105	α-Thujone	tr	---	tr	---	0.1	0.1	tr	tr	---	---
1112	1112	(*E*)-2,4-Dimethylhepta-2,4-dienal	0.1	---	tr	---	0.2	0.2	---	---	---	0.2
1113	1113	Phenethyl alcohol	---	---	tr	---	---	---	---	---	---	---
1118	1118	β-Thujone	0.2	0.1	0.1	---	0.6	1.0	0.1	0.5	tr	0.6
1119	1120	*endo*-Fenchol	---	---	tr	0.1	---	---	---	---	0.1	---
1119	1118	Dehydrosabina ketone	---	---	---	---	---	---	---	0.1	---	---
1121	1122	Chrysanthenone	---	---	---	---	---	0.1	---	---	0.3	0.1
1122	1122	*trans-p*-Mentha-2,8-dien-1-ol	0.2	0.7	0.3	tr	0.4	tr	0.1	0.1	---	---
1124	1124	*cis-p*-Menth-2-en-1-ol	---	---	---	---	0.1	0.1	0.3	0.1	tr	0.1
1127	1127	α-Campholenal	---	---	0.3	0.3	0.9	0.9	0.1	0.5	1.2	0.7
1132	1132	*cis*-Limonene oxide	1.2	0.7	0.1	tr	0.2	tr	tr	0.1	---	---
1136	1137	*trans*-Limonene oxide	---	---	---	0.1	---	---	---	---	---	---
1137	1137	*cis-p*-Mentha-2,8-dien-1-ol	0.2	1.2	0.4	---	0.4	---	0.2	---	0.1	tr
1138	1139	Nopinone	---	---	---	0.1	0.3	0.5	tr	0.3	0.2	0.2
1140	1140	*trans*-Sabinol	0.1	---	---	---	---	---	---	---	---	---
1141	1141	*trans*-Pinocarveol	0.1	1.4	0.9	0.8	3.8	4.4	0.7	5.0	3.5	2.7
1141	1141	*cis*-Verbenol	---	0.1	---	0.3	0.7	0.4	---	---	0.7	0.6
1143	1142	*trans-p*-Menth-2-en-1-ol	0.2	---	---	---	---	---	0.3	---	---	---
1146	1145	*trans*-Verbenol	0.1	4.3	2.0	1.6	8.0	9.1	0.8	11.2	7.0	5.5
1147	1145	Camphor	---	---	---	0.1	---	---	tr	---	---	---
1149	1149	*cis*-β-Terpineol	---	tr	---	---	---	---	---	---	---	---
1149	1154	*trans-p*-Isopropylcyclohexanol	---	---	---	---	---	---	0.1	---	---	---
1150	1150	α-Phellandren-8-ol	1.0	0.1	0.1	0.2	0.2	0.1	0.1	0.1	0.3	0.3
1157	1157	Sabina ketone	---	0.1	tr	tr	0.2	0.3	tr	0.2	0.2	0.3
1160	1160	*trans*-Pinocamphone	---	0.2	0.1	0.1	0.5	0.6	0.1	0.5	0.5	0.2
1162	1164	Pinocarvone	---	0.2	0.1	0.2	0.5	0.6	0.1	0.7	0.3	0.4
1169	1168	α-Phellandrene epoxide	0.2	0.1	0.1	---	0.4	0.6	0.1	0.3	---	0.4
1171	1171	*p*-Mentha-1,5-dien-8-ol	0.6	0.1	0.2	0.6	0.6	---	0.2	0.4	1.0	0.7
1173	1173	Borneol	---	---	---	---	---	0.5	0.1	---	---	---
1176	1176	*cis*-Pinocamphone	---	---	---	tr	0.2	0.2	tr	0.2	0.1	0.1
1178	1179	2-Isopropenyl-5-methyl-4-hexenal	0.3	0.2	0.1	---	0.1	tr	tr	---	---	---
1180	1180	Terpinen-4-ol	---	0.4	1.6	1.6	1.3	1.5	0.9	1.3	0.6	0.8
1182	1178	*m*-Cymen-8-ol	3.9	---	---	---	---	---	---	---	---	---
1183	1183	Thuj-3-en-10-al	---	---	---	---	---	0.1	---	---	---	---
1185	1188	*p*-Methylacetophenone	0.1	0.1	0.1	tr	0.2	0.1	0.2	0.1	0.1	---
1186	1186	Cryptone	---	---	---	---	---	---	0.6	---	---	---
1187	1189	*p*-Cymen-8-ol	1.8	0.5	0.7	0.3	1.7	1.3	1.3	1.0	0.9	0.4
1193	1194	*p*-Mentha-1,5-dien-7-ol	---	---	---	---	---	0.1	---	---	---	---
1194	---	5-Isopropenyl-2-methyl-7-oxabicyclo[4.1.0]heptan-2-ol	0.4	---	---	---	---	---	---	---	---	---
1195	1195	α-Terpineol	0.3	---	2.0	2.3	2.3	---	2.6	---	1.0	---
1196	1196	Myrtenal	---	0.6	---	---	---	2.2	---	2.1	---	0.7
1197	1195	Myrtenol	---	0.6	0.2	0.4	1.2	2.0	---	1.3	0.7	1.3
1198	1198	*trans*-Dihydrocarvone	0.1	0.1	0.1	---	---	---	---	---	0.1	---
1201	1201	*cis*-Piperitenol	0.1	0.2	0.2	tr	0.1	---	0.1	---	---	---
1204	1202	*cis*-Sabinol	0.2	---	0.6	0.1	0.2	0.1	1.8	---	---	---
1208	1208	Verbenone	---	1.0	0.3	0.8	2.7	3.9	0.3	4.8	1.7	0.7
1210	1209	*trans*-Piperitol	tr	---	---	tr	---	---	0.3	---	---	---
1212	1211	4-Methyleneisophorone	0.3	---	---	---	---	---	---	0.1	---	---
1219	1218	*trans*-Carveol	0.2	2.0	1.0	0.3	1.9	1.2	0.5	1.2	1.6	0.6
1223	1223	*p*-Cumenol	---	---	---	---	---	---	0.1	---	---	---
1226	1222	2-Hydroxycineole	---	---	---	---	---	---	0.1	---	---	---
1230	1230	*cis-p*-Mentha-1(7),8-dien-2-ol	0.1	0.1	0.1	---	---	---	0.1	---	---	---
1233	1232	*cis*-Carveol	0.1	0.5	0.2	0.1	0.3	0.1	0.2	0.1	---	---
1242	1242	Cuminal	---	---	0.1	tr	0.2	0.1	0.5	0.1	---	---
1244	1246	Carvone	0.2	1.6	0.5	0.1	0.8	0.3	0.2	0.3	0.3	0.1
1249	1249	Car-3-en-2-one	0.3	---	---	---	---	---	---	---	---	---
1249	1249	Carvotanacetone	---	---	0.3	---	0.2	0.2	0.9	0.1	0.1	0.2
1251	1250	Linalyl acetate	0.2	0.1	---	5.1	---	---	---	---	---	---
1252	1252	Pinocamphone	---	---	---	---	---	0.1	---	---	---	---
1253	1254	2-Hydroxypinocamphone	---	---	---	---	---	---	---	tr	---	---
1254	1254	Piperitone	---	---	---	---	---	0.1	0.3	---	---	---
1270	1270	*iso*-Piperitenone	---	0.1	tr	---	---	---	---	---	---	---
1271	1271	2,4-Dimethylphenethyl alcohol	---	---	---	---	---	---	0.1	---	---	---
1274	1276	2,3-Pinanediol	---	0.2	0.1	0.1	0.2	0.2	---	0.2	0.2	0.2
1276	1277	Perilla aldehyde	---	0.1	0.1	tr	0.2	0.1	---	0.1	---	---
1277	1277	*trans*-Carvone oxide	---	0.1	0.1	---	---	---	---	---	---	---
1278	1277	Phellandral	---	---	---	0.1	0.1	0.1	0.2	tr	---	---
1285	1285	Bornyl acetate	0.1	0.2	0.3	0.3	0.8	0.8	0.4	0.6	tr	0.7
1291	1291	Safrole	---	---	---	---	---	---	---	---	0.7	---
1292	1293	Thymol	0.3	0.2	0.1	---	0.3	0.2	0.3	0.1	---	0.2
1292	1291	*p*-Cymen-7-ol	---	---	0.2	0.1	0.4	0.1	0.5	0.1	---	---
1299	1300	Carvacrol	0.3	---	0.6	0.1	0.7	0.7	1.1	0.3	---	---
1300	1299	Perilla alcohol	---	0.1	tr	---	---	---	---	---	---	---
1311	1314	Car-3-en-5-one	0.7	---	---	---	---	---	---	---	---	---
1317	1316	Filifolide A	---	---	---	---	---	---	---	---	0.1	tr
1320	1318	3-Hydroxycineole	0.1	---	0.2	0.1	---	---	0.2	---	---	---
1320	1324	1-Hydroperoxy-*p*-mentha-2,8-diene	---	0.3	---	---	---	---	---	---	---	---
1346	1346	Limonene-1,2-diol	---	0.1	---	---	---	---	---	---	---	---
1348	1349	α-Terpinyl acetate	0.9	0.3	0.4	0.3	0.4	0.2	1.0	---	---	---
1349	1349	α-Cubebene	---	---	---	---	---	---	---	0.1	---	0.1
1352	1352	α-Longipinene	---	---	---	0.1	0.1	0.1	---	0.2	---	---
1359	---	6-Hydroperoxy-*p*-mentha-1,8-diene	---	0.3	---	---	---	---	---	---	---	---
1360	1361	Neryl acetate	---	---	---	0.1	---	---	---	---	---	---
1376	1378	β-Sinensal	---	0.2	---	---	---	---	---	---	---	---
1377	1372	Longicyclene	---	---	---	0.1	---	---	---	---	---	---
1377	1377	α-Copaene	---	---	0.2	---	1.0	2.0	tr	0.8	0.1	3.0
1380	1380	Geranyl acetate	---	---	---	0.1	---	---	---	---	---	---
1384	1383	*cis*-β-Elemene	---	---	---	---	---	---	---	---	---	tr
1389	1387	β-Cubebene	---	---	---	---	---	---	---	---	---	0.1
1391	1390	*trans*-β-Elemene	---	0.1	0.4	---	1.8	2.8	0.2	2.2	1.8	5.7
1401	1403	Methyl eugenol	---	---	---	---	---	---	---	---	0.1	---
1407	1407	Cyperene	---	---	---	0.1	---	---	---	---	---	---
1456	1452	(*E*)-β-Farnesene	---	---	---	0.1	---	---	---	---	---	---
1460	1460	*allo*-Aromadendrene	---	---	---	---	0.1	0.2	---	0.1	---	0.2
1475	1475	γ-Muurolene	---	---	---	---	---	0.1	---	---	---	0.1
1489	1489	β-Selinene	---	---	tr	---	0.1	0.1	---	tr	0.1	0.2
1493	1487	4,10-Epoxyamorphane	---	---	---	0.2	---	---	---	---	---	---
1498	1497	α-Selinene	---	---	tr	tr	tr	0.1	---	---	tr	0.1
1499	1500	α-Muurolene	---	---	---	---	---	tr	---	---	---	0.1
1505	1504	α-Cuprenene	---	---	---	0.2	---	---	---	---	---	---
1515	1514	γ-Cadinene	---	---	---	---	0.2	0.2	---	---	---	---
1518	1519	Cubebol	---	---	---	---	0.1	0.1	---	---	---	---
1520	1520	δ-Cadinene	---	---	tr	---	0.1	0.1	---	---	---	0.1
1578	1578	Spathulenol	---	---	---	---	0.1	0.1	---	0.1	---	0.1
1585	1587	Caryophyllene oxide	---	---	---	---	0.1	0.2	---	---	tr	0.1
1591	1591	β-Copaen-4α-ol	---	---	---	---	tr	0.1	---	tr	---	---
1605	1605	Ledol	---	---	---	---	---	tr	---	tr	---	---
1611	1611	Humulene epoxide II	---	---	---	---	tr	tr	---	tr	---	---
1614	1618	β-Himachalene oxide	---	---	---	---	tr	tr	---	tr	---	---
1617	1616	1,10-di-*epi*-Cubenol	---	---	---	---	tr	0.1	---	---	---	---
1644	1643	τ-Cadinol	---	---	---	---	0.3	0.4	tr	tr	---	---
1645	1645	τ-Muurolol	---	---	---	---	tr	tr	tr	tr	---	---
1675	1676	Mustakone	---	---	---	---	0.1	0.2	---	0.1	---	---
1726	1726	α-Phellandrene dimer	---	---	---	---	0.1	---	tr	---	---	---
1734	1739	α-Phellandrene dimer	tr	---	---	---	---	---	0.2	---	---	---
1769	1769	Benzyl benzoate	---	---	---	---	---	---	---	tr	0.1	---
1794	1793	α-Phellandrene dimer	1.0	0.1	0.3	0.1	1.9	0.7	4.0	0.2	---	---
1800	1797	α-Phellandrene dimer	0.1	tr	tr	tr	0.2	0.1	0.6	tr	---	---
1811	1810	α-Phellandrene dimer	tr	---	---	---	tr	---	0.1	---	---	---
1826	1825	α-Phellandrene dimer	tr	tr	tr	tr	tr	0.1	0.1	tr	---	---
1829	1829	α-Phellandrene dimer	0.1	---	---	---	0.3	---	0.6	---	---	---
1832	---	α-Phellandrene dimer	tr	---	---	tr	tr	---	0.1	tr	---	---
1912	1913	α-Phellandrene dimer	tr	---	---	tr	tr	---	0.1	---	---	---
1929	1929	α-Phellandrene dimer	tr	---	---	tr	tr	---	0.1	---	---	---
1949	1951	(3*E*)-Cembrene A	---	---	tr	---	---	---	---	---	---	---
2023	2022	Manoyl oxide	---	---	---	---	---	---	---	tr	---	---
2130	2127	Neocembrene A	---	---	tr	---	---	---	---	---	---	---
2143	2143	Serratol	---	tr	tr	---	tr	---	tr	---	---	---
		Monoterpene hydrocarbons	81.1	75.1	82.8	81.1	56.0	51.5	73.5	59.2	71.1	67.2
		Oxygenated monoterpenoids	15.4	19.5	14.9	17.8	35.7	37.1	18.2	34.6	22.9	18.9
		Sesquiterpene hydrocarbons	0.0	0.1	0.6	0.6	3.3	5.6	0.2	3.3	1.9	9.7
		Oxygenated sesquiterpenoids	0.0	0.2	tr	0.2	0.5	1.2	tr	0.2	tr	0.2
		Diterpenoids	1.3	0.1	0.3	0.1	2.5	1.0	5.7	0.2	0.0	0.0
		Benzenoid aromatics	0.2	0.2	0.4	tr	0.4	0.2	0.6	0.1	1.6	0.3
		Others	0.4	0.0	0.1	tr	0.6	0.8	0.1	0.4	0.5	1.2
		Total identified	98.4	95.1	99.1	99.7	99.1	97.4	98.4	98.1	98.1	97.5

RI_calc_ = retention index determined with respect to a homologous series of *n*-alkanes on a ZB-5ms column. RI_db_ = retention index obtained from the databases. Samples A–C are pure *Boswellia rivae*; samples D–H are commercial samples collected from villages; samples I and J are pure *Commiphora africana* (see Table 3). tr = trace (<0.05%).

**Table 3:** Enantiomeric distribution of chemical components in *B. rivae* and *C. africana* resin essential oils.

Compound	RT_std_	RT_EO_	A	B	C	D	E	F	G	H	I	J
(+)-α-Thujene	13.918	nd	0.0	0.0	0.0	0.0	0.0	0.0	0.0	0.0	---	0.0
(–)-α-Thujene	13.992	13.929	100	100	100	100	100	100	100	100	---	100
(–)-α-Pinene	15.915	15.587	24.1	0.3	43.5	94.8	72.4	96.8	17.4	96.5	0.2	95.2
(+)-α-Pinene	16.402	16.284	75.9	99.7	56.5	5.2	27.6	3.2	82.6	3.5	99.8	4.8
(–)-Camphene	17.733	18.001	---	31.1	78.5	92.4	90.5	96.7	67.4	95.9	52.4	95.6
(+)-Camphene	18.300	18.411	---	68.9	21.5	7.6	9.5	3.3	32.6	4.1	47.6	4.4
(+)-Sabinene	19.740	19.729	20.0	19.9	20.9	49.9	39.5	48.2	20.3	37.0	---	21.8
(–)-Sabinene	20.603	20.530	80.0	80.1	79.1	50.1	60.5	51.8	79.7	63.0	---	78.2
(+)-β-Pinene	20.271	20.301	48.0	97.0	14.3	2.1	4.1	2.3	33.9	2.5	98.9	4.4
(–)-β-Pinene	20.625	20.803	52.0	3.0	85.7	97.9	95.9	97.7	66.1	97.5	1.1	95.6
(+)-δ-3-Carene	22.737	22.949	100	100	100	---	100	100	100	100	---	100
(–)-δ-3-Carene	na	nd	0.0	0.0	0.0	---	0.0	0.0	0.0	0.0	---	0.0
(–)-α-Phellandrene	22.589	22.584	---	---	---	5.5	0.0	0.0	0.0	---	---	---
(+)-α-Phellandrene	22.812	22.774	---	---	---	94.5	100	100	100	---	---	---
(–)-Limonene	25.061	25.120	43.7	2.6	7.0	67.7	20.4	60.2	41.0	47.2	38.7	44.2
(+)-Limonene	25.992	25.897	56.3	97.4	93.0	32.3	79.6	39.8	59.0	52.8	61.3	55.8
(–)-β-Phellandrene	26.150	26.333	---	---	---	14.5	---	---	---	---	---	---
(+)-β-Phellandrene	26.881	26.689	---	---	---	85.5	---	---	---	---	---	---
(+)-*cis*-Sabinene hydrate	40.701	40.781	18.3	---	17.3	---	22.5	20.2	---	22.1	---	---
(–)-*cis*-Sabinene hydrate	41.252	41.291	81.7	---	82.7	---	77.5	79.8	---	77.9	---	---
(–)-Linalool	45.691	45.731	---	---	---	33.4	---	---	50.2	---	---	---
(+)-Linalool	46.241	46.226	---	---	---	66.6	---	---	49.8	---	---	---
(+)-β-Thujone	46.057	46.008	100	---	---	---	---	---	---	100	---	---
(–)-β-Thujone	na	nd	0.0	---	---	---	---	---	---	0.0	---	---
(+)-*trans*-Sabinene hydrate	46.155	46.210	---	---	---	---	---	---	---	22.7	---	---
(–)-*trans*-Sabinene hydrate	46.838	46.878	---	---	---	---	---	---	---	77.3	---	---
(–)-Camphor	49.315	50.308	---	---	---	---	---	---	---	---	79.4	---
(+)-Camphor	50.125	50.570	---	---	---	---	---	---	---	---	20.6	---
(+)-Terpinen-4-ol	54.635	54.688	19.7	25.9	12.4	30.7	21.8	20.4	37.9	16.6	74.4	35.5
(–)-Terpinen-4-ol	54.932	55.021	80.3	74.1	87.6	69.3	78.2	79.6	62.1	83.4	25.6	64.5
(–)-α-Terpineol	59.734	59.734	28.0	7.6	22.8	84.4	35.4	84.3	9.8	84.9	0.0	83.2
(+)-α-Terpineol	60.576	60.495	72.0	92.4	77.2	15.6	64.6	15.7	90.2	15.1	100	16.8
(+)-Verbenone	61.700	61.788	---	100	---	100	100	100	---	100	---	---
(–)-Verbenone	na	nd	---	0.0	---	0.0	0.0	0.0	---	0.0	---	---
(–)-α-Copaene	62.855	62.855	---	---	---	---	---	---	---	---	---	100
(+)-α-Copaene	na	nd	---	---	---	---	---	---	---	---	---	0.0
(+)-Carvone	62.910	62.910	---	49.5	---	---	---	---	---	---	---	---
(–)-Carvone	63.065	63.065	---	50.5	---	---	---	---	---	---	---	---
(–)-*trans*-β-Elemene	66.129	66.163	---	---	---	---	---	---	---	100	100	100
(+)-*trans*-β-Elemene	na	nd	---	---	---	---	---	---	---	0.0	0.0	0.0

RT_std_ = retention time (min) of the standard compounds from Sigma-Aldrich. RT_EO_ = average retention time of the essential oil component. na = standard compound not available. nd = enantiomer not detected.

## Data Availability

Data are available from the corresponding authors (W.N.S. or S.J.) upon reasonable request.
